# Atrial Septal Aneurysm and Anxiety: A Matched Cross-Sectional Study with Prospective Enrollment Exploring Clinical and Psychosomatic Associations

**DOI:** 10.3390/jcm15208046

**Published:** 2026-10-16

**Authors:** Doğaç Çağlar Gürbüz, Hasan Atmaca, Hicran Damar, Mustafa Kemal Erol, Ertan Yetkin

**Affiliations:** 1 Department of Cardiology, Şişli Kolan International Hospital, 34381 Istanbul, Türkiye; 2 Department of Cardiology, Acıbadem Taksim Hospital, 34373 Istanbul, Türkiye; 3 Department of Cardiology, Üsküdar University, 34662 Istanbul, Türkiye; 4 Department of Cardiology, Türkiye Hospital, 34381 Istanbul, Türkiye; 5 Department of Neurology, Faculty of Medicine, Alaaddin Keykubat University, 07425 Alanya, Türkiye; 6 Department of Cardiology, Faculty of Medicine, Istanbul Health and Technology University, 34445 Istanbul, Türkiye

**Keywords:** palpitation, echocardiography, atrial septal aneurysm (ASA), anxiety, State–Trait Anxiety Inventory (STAI), Beck Depression Inventory (BDI)

## Abstract

**Background/Objectives:** An isolated atrial septal aneurysm (ASA) represents a distinct structural anomaly defined by abnormal bulging of the interatrial tissue, affecting roughly three percent of the general public. Although routinely viewed as an accidental discovery during imaging, this condition carries recognized associations with adverse clinical events, notably cerebrovascular ischemia and rhythm disturbances. Because the interplay between cardiovascular diseases and psychological distress is well established, our primary goal was to determine if individuals harboring this septal anomaly display elevated symptoms of anxiety or depression relative to standard cardiology outpatients. **Methods**: For this comparative cross-sectional investigation, we recruited a total of one hundred and twenty adults from a dedicated cardiology clinic. The cohort consisted of sixty individuals diagnosed with the septal anomaly and sixty healthy control subjects, carefully paired according to age and gender. Every participant received a comprehensive clinical baseline assessment, which included a 12-lead electrocardiogram alongside a detailed two-dimensional transthoracic echocardiogram. Psychological status was assessed using the State–Trait Anxiety Inventory (STAI-1 for state anxiety and STAI-2 for trait anxiety) and the Beck Depression Inventory (BDI). **Results:** Initial physical and demographic profiling—specifically regarding patient age, gender distribution, and calculated body mass indices—revealed no significant statistical variances between the two study arms. The ASA group presented with a significantly higher prevalence of clinical palpitations (83.3% vs. 43.3%, *p* < 0.001). Furthermore, the ASA group exhibited significantly higher scores on all psychometric measures (STAI-1: 44.97 ± 9.15 vs. 32.53 ± 8.24, *p* < 0.001; STAI-2: 49.37 ± 7.22 vs. 34.72 ± 10.55, *p* < 0.001; BDI: 19.66 ± 9.79 vs. 10.07 ± 6.81, *p* < 0.001). After confirming the absence of severe multicollinearity (Variance Inflation Factor < 5), exploratory logistic regression analysis demonstrated that clinical palpitations (odds ratio [OR]: 5.309; 95% confidence interval [CI]: 1.621–17.383; *p* = 0.006) and trait anxiety scores (STAI-2) (OR: 1.169 per one-point increment; 95% CI: 1.062–1.286; *p* = 0.001) were independently associated factors for the presence of ASA. **Conclusions:** This study demonstrates a significant clinical association between ASA, clinical palpitations, and persistent trait anxiety (STAI-2). While the cross-sectional design precludes causal inferences, the findings suggest a clinical clustering of anxiety and palpitations in patients diagnosed with ASA. This highlights the importance of recognizing potential psychosomatic overlaps in structural heart diseases.

## 1. Introduction

Atrial septal aneurysm (ASA) is characterized by protrusion of the interatrial septum into either the left or right atrium, with a base and protrusion width exceeding 15 mm in the fossa ovalis region [1,2]. Although ASA is generally considered a benign cardiac anomaly with a prevalence of 3%, it has been linked to clinical outcomes such as cryptogenic stroke and paroxysmal atrial fibrillation [2,3,4]. Embryologically, ASA is associated with the septum primum, which forms the base and is encircled by the limbs of the septum primum and the secundum. The condition is marked by redundant, mobile, and thin tissue bulging into the atria, and it has also been linked to other structural cardiac pathologies, including mitral valve prolapse (MVP) and regurgitation [5,6]. The association between cardiovascular diseases and psychological conditions, such as anxiety and mood disorders, has been extensively documented, establishing a strong bidirectional relationship [7,8]. In structurally mild or silent heart diseases, the literature has primarily focused on the relationship between cardiac abnormalities and anxiety in the context of MVP [9]. However, the psychosomatic aspects of other prevalent anomalies like ASA remain underexplored. Therefore, this study aimed to evaluate the clinical association between the presence of ASA and standardized measures of anxiety and depression in a cardiology outpatient cohort.

## 2. Materials and Methods

### 2.1. Study Population, Setting, and Ethical Approval

**Figure 1:**
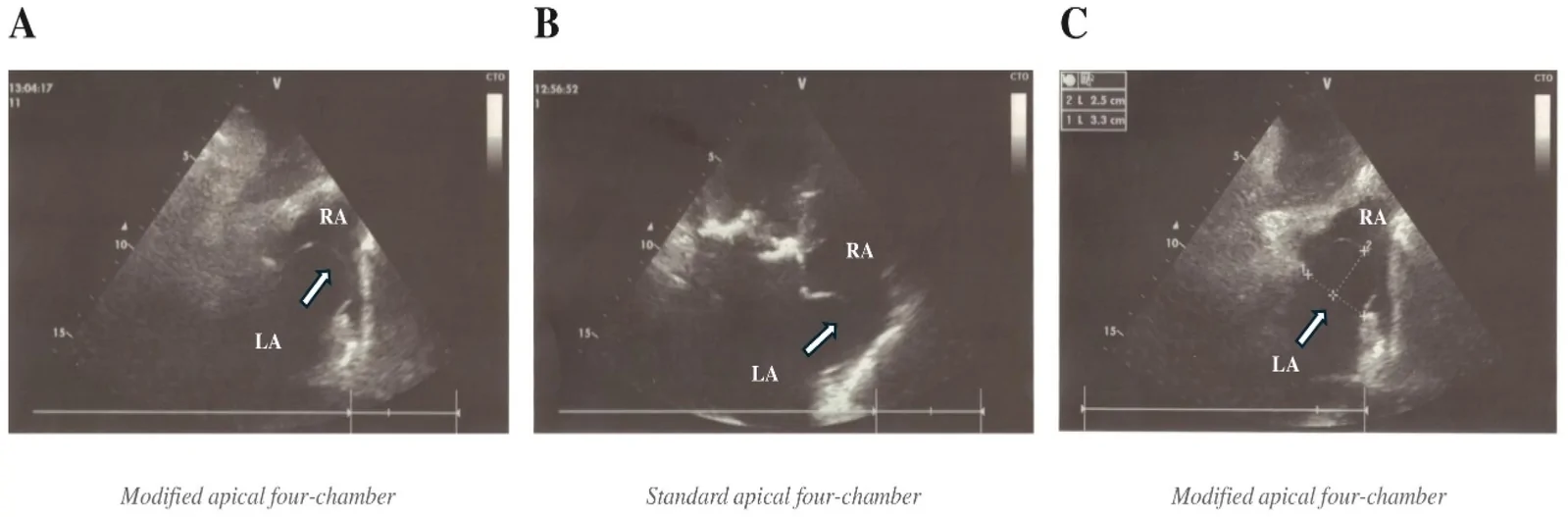
Representative transthoracic echocardiography images of an atrial septal aneurysm (ASA). Panels (**A**,**C**) display modified apical 4-chamber views, while Panel (**B**) represents a standard apical 4-chamber view. The white arrows in Panels (**A**,**B**) indicate the widest points of the aneurysmal sac protruding into the atrium, demonstrating its distinct structural morphology during the cardiac cycle. In Panel (**C**), the white arrow highlights the diagnostic measurement calipers, explicitly indicating a base width of 3.3 cm and a maximal septal excursion of 2.5 cm, strictly fulfilling the established diagnostic criteria for ASA.

**Figure 2:**
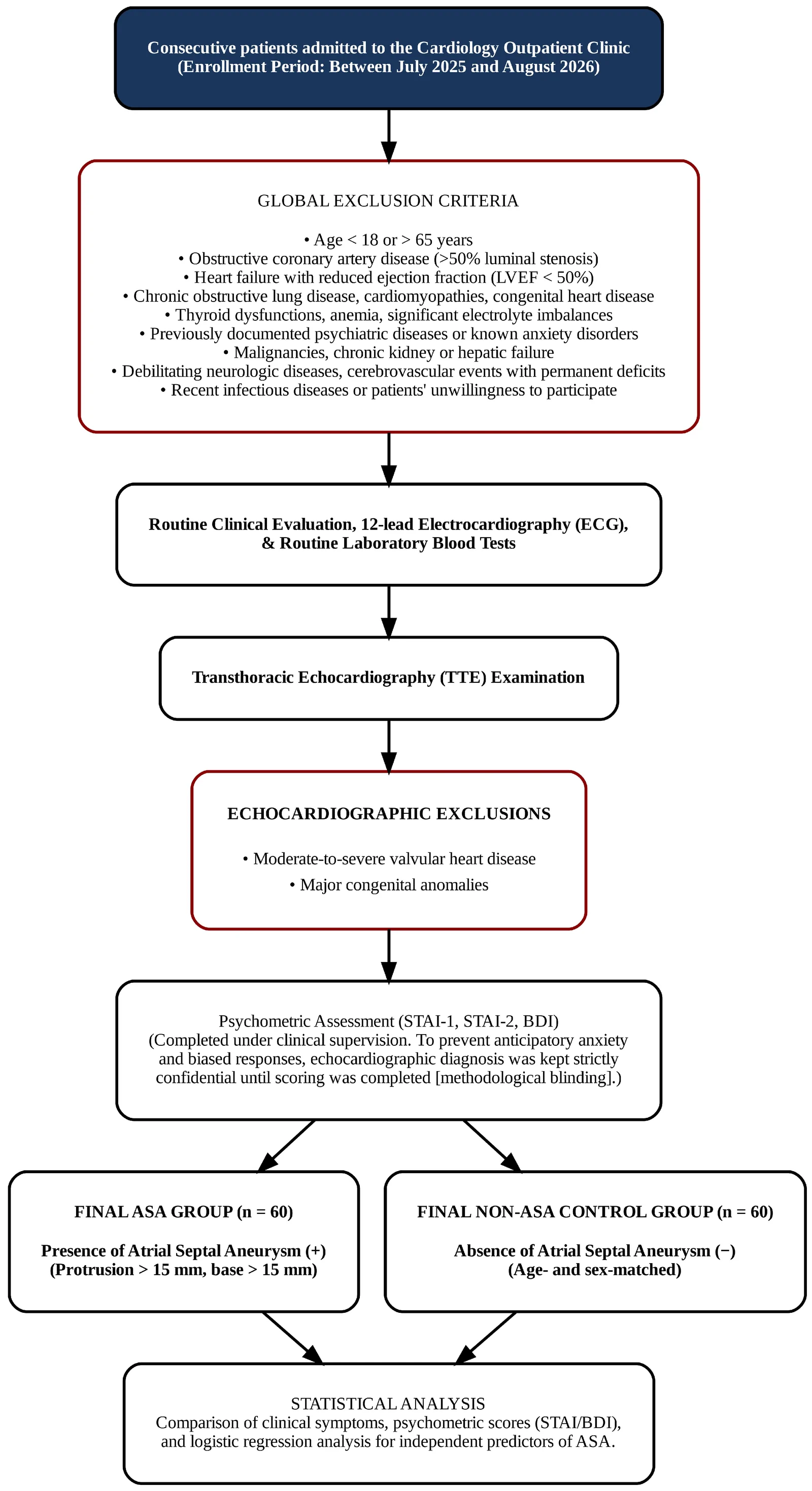
Enrollment flowchart and study methodology detailing patient selection, global exclusion criteria, and the blinded sequence of clinical, echocardiographic, and psychometric assessments. While eligible participants were enrolled consecutively, exact numerical tracking of globally screened but excluded routine outpatients was not systematically recorded; therefore, absolute exclusion numbers are not presented. The echocardiographic diagnosis was strictly withheld from participants until psychometric scoring was completed to prevent anticipatory anxiety.

We designed a matched, cross-sectional observational model involving 120 adult participants aged 18 to 65. The cohort was strictly divided into sixty individuals harboring the septal anomaly (illustrative ultrasound captures are provided in Figure 1) and sixty control subjects without an ASA. To ensure rigorous methodology, chronological age and gender matching was executed on a strict 1:1 individual basis. Furthermore, these control individuals were specifically recruited from the exact same outpatient clinic, presenting with comparable non-specific physical complaints (primarily routine cardiac screening, atypical chest discomfort, and clinical palpitations) but demonstrating completely normal structural echocardiograms. Prospective patient enrollment and data collection were conducted consecutively at the specialized cardiology outpatient department of Private Şişli Kolan Hospital spanning from July 2025 through August 2026. While we consecutively enrolled eligible patients, we did not systematically track the exact number of all screened general outpatients who were globally excluded; therefore, absolute screening numbers cannot be provided. Crucially, none of the participants were previously aware of their ASA diagnosis; it was identified incidentally during the current evaluation (Figure 2).

While clinical patient enrollment and consecutive data collection were conducted exclusively at Private Şişli Kolan Hospital in Istanbul, the study protocol was formally reviewed and approved by the Non-Interventional Clinical Research Ethics Committee of Alaaddin Keykubat University in Alanya (protocol code: 11-27; date of approval: 25 June 2025). This institutional ethical arrangement was specifically established because Alaaddin Keykubat University served as the coordinating academic center for our co-investigator from the Department of Neurology, who provided expert oversight for the collaborative evaluation of the psychometric and neurological data. Written informed consent was obtained from all study participants prior to clinical and psychometric evaluation.

### 2.2. Clinical Protocol and Psychometric Assessment

Upon admission to the outpatient clinic, following standard demographic data collection, all participants first underwent 12-lead electrocardiography and transthoracic echocardiography as part of their routine evaluation. Immediately following the echocardiographic examination, patients were asked to complete the State and Trait Anxiety Inventory (STAI-1 [STAI-State] and STAI-2 [STAI-Trait]) and the Beck Depression Inventory (BDI) under clinician supervision [10,11,12]. The validated Turkish versions of the STAI and BDI were administered to the participants. Any questionnaires with missing or incomplete responses were planned for exclusion from the final analysis; however, all 120 enrolled participants provided complete questionnaire datasets [13,14]. Crucially, the echocardiographic diagnosis (i.e., the presence or absence of an ASA) was strictly withheld from the patients until they had fully completed the questionnaires. This methodological blinding to their structural cardiac status was deliberately implemented to prevent any anticipatory anxiety or biased responses on the psychometric scales. Once all questionnaires were completed and collected, the patients were subsequently informed of their echocardiographic findings in detail.

### 2.3. Exclusion Criteria

Individuals were disqualified from participation if their clinical workup revealed significant coronary vessel blockages (luminal narrowing greater than fifty percent), impaired left ventricular systolic function (ejection fraction dropping below fifty percent), or advanced pulmonary disorders. We further excluded anyone diagnosed with structural cardiomyopathies, severe congenital cardiac malformations, or active thyroid disease. Additional disqualifying conditions included a formally documented history of psychiatric illnesses or established anxiety syndromes, active oncological conditions, end-stage renal or liver dysfunction, severe neurological impairment, residual deficits from prior strokes, profound blood cell count abnormalities, major electrolyte shifts, acute systemic infections, or those declining informed consent.

### 2.4. Demographic Data and Clinical Definitions

The diagnostic benchmark for hypertension required repeated resting blood pressure measurements of 140/90 mmHg or greater, or the ongoing daily use of blood pressure-lowering therapeutics. We established the presence of diabetes mellitus if a subject demonstrated a fasting plasma glucose reading of at least 126 mg/dL, a random glucose test reaching 200 mg/dL alongside typical clinical symptoms, or active reliance on hypoglycemic agents. Subjects met the criteria for hyperlipidemia if their total serum cholesterol measurements exceeded 200 mg/dL, low-density lipoprotein profiles were higher than 130 mg/dL, or if they were currently prescribed lipid-lowering statin drugs. Routine anthropometric calculations for body mass index (BMI) were completed for every enrolled subject. A depressed left ventricular ejection fraction, serving strictly as a primary threshold for study exclusion rather than a generalized clinical label, was classified as any measurement falling beneath the fifty percent mark. Transthoracic echocardiography was performed by an experienced cardiologist using the Philips Affinity 70 Ultrasound System (Philips Healthcare, Amsterdam, The Netherlands). Standard imaging measurements, including aortic root, left atrial, and left ventricular dimensions, were obtained according to the American Society of Echocardiography and European Association of Cardiovascular Imaging guidelines for chamber quantification (Lang RM, et al.) [15]. ASA was defined as the protrusion of the interatrial septum more than 15 mm into either atrium or a phasic excursion exceeding 15 mm during the respiratory cycle, with a base diameter greater than 15 mm (>15 mm). Furthermore, patients found to have moderate-to-severe valvular heart disease or major congenital anomalies during the transthoracic echocardiography examination were excluded from the study.

### 2.5. Statistical Analyses

Prior to data collection, the minimum sample size was projected at 27 subjects per group to perform an independent samples *t*-test. This computation was executed via G*Power software (version 3.1; Heinrich-Heine-Universität Düsseldorf, Düsseldorf, Germany), adopting an alpha error probability of 0.05 and a statistical power of 95% (1-beta). Our baseline assumptions aimed to identify a clinically significant variance in the primary outcomes (mean STAI and BDI scores). Specifically, drawing upon expected variability metrics from comparable structural cardiac anomalies—notably mitral valve prolapse—this calculation was powered to detect a large standardized effect size (Cohen’s d = 0.8), anchored to a baseline expectation of at least a 20% clinical variance in psychometric profiles as previously reported [9]. While this initial calculation guaranteed sufficient power for the primary intergroup comparisons, we recognize it does not inherently validate the statistical power for all secondary exploratory outcomes or the multivariable regression model. Consequently, we expanded our final analytical cohort to 60 individuals per group (total *n* = 120) to ensure a more robust clinical evaluation. All quantitative data management and subsequent predictive modeling tasks were processed using SPSS software, release 27.0 (IBM Corp., Armonk, NY, USA). To verify the distribution patterns of our continuous datasets, we ran a combination of Shapiro–Wilk and Kolmogorov–Smirnov statistical evaluations. Continuous clinical parameters that followed a normal distribution curve are expressed as central mean values accompanied by standard deviations, whereas categorical observations are presented as raw counts and corresponding group percentages. When comparing numerical continuous data between our two distinct study groups, we relied upon the independent samples *t*-test. Proportional differences within our categorical variables were cross-examined using Pearson’s Chi-square methodology, substituting with Fisher’s exact test whenever expected cell count assumptions were not met. Given the multiplicity of variables analyzed, these secondary comparative tests should be viewed as exploratory. Before building the regression model, we tested for potential multicollinearity among the psychological assessment scores by calculating Variance Inflation Factors (VIFs). A VIF threshold strictly below 5 was established to rule out severe multicollinearity, thereby permitting the simultaneous integration of these psychometric variables into the model. Finally, an exploratory multivariable logistic regression framework was constructed to identify independent predictors, designating the structural diagnosis of an atrial septal aneurysm as the primary dependent target variable. The selection of variables for inclusion in this multivariable model was strategically based on their fundamental clinical relevance coupled with a preliminary univariate significance threshold of *p* < 0.05. To maintain model parsimony given the sample size, standard demographic covariates such as age and gender—which were inherently controlled for via strict 1:1 matching—and body mass index were not forced into the primary model; however, a subsequent sensitivity analysis adjusting for these demographic factors confirmed the robustness of the primary findings. The final outcomes are presented as Odds Ratios (ORs) with their respective 95% Confidence Intervals (CIs), and any two-sided *p*-value dropping below the 0.05 threshold was deemed statistically significant.

## 3. Results

### 3.1. Baseline Demographic and Clinical Characteristics

The expanded study cohort included 60 patients with ASA and 60 non-ASA cardiology outpatients (Table 1, Supplementary Figure S1). Baseline demographic parameters, including mean age (39.78 ± 12.03 vs. 37.43 ± 8.94 years; *p* = 0.227) and female gender distribution (33.3% vs. 35.0%; *p* = 0.849), were well-matched. No significant differences were observed in cardiovascular risk factors such as hypertension, diabetes mellitus, hyperlipidemia, or smoking status (*p* > 0.05 for all). Anthropometric measurements, particularly BMI (25.90 ± 4.19 vs. 25.23 ± 4.07 kg/m^2^; *p* = 0.379), were comparable. Furthermore, routine laboratory parameters that could secondarily induce palpitations (e.g., hemoglobin levels, serum electrolytes, and thyroid function tests) were systematically evaluated for all participants, revealing no statistically significant intergroup differences. Regarding clinical presentation, the prevalence of palpitations was significantly higher in the ASA group compared to the control group (83.3% vs. 43.3%; *p* < 0.001). Regarding neurological history, 2 patients (3.3%) in the ASA group had a history of transient ischemic attack (TIA) and amaurosis fugax, whereas none were observed in the control group. A history of migraine was present in 1 patient (1.7%) in the ASA group and 2 patients (3.3%) in the control group (*p* = 0.563). All patients with neurological symptoms or history had been formally evaluated and cleared by a neurology specialist prior to study inclusion.

### 3.2. Electrocardiographic and Echocardiographic Parameters

Electrocardiographic findings, including baseline heart rate (77.17 ± 12.54 vs. 76.30 ± 11.19 bpm; *p* = 0.691), PR interval, QRS duration, and QTc intervals, showed no statistically significant intergroup differences (Table 2). Furthermore, 24 h rhythm Holter monitoring was performed in a highly symptomatic subset of 22 participants (13 patients in the ASA group and 9 in the control group) who presented with prominent clinical palpitations. While no clinically significant arrhythmias were detected in these specific recordings, we acknowledge that this limited monitoring does not completely exclude the potential presence of uncaptured paroxysmal arrhythmias throughout the broader unmonitored cohort. On transthoracic echocardiography (Table 3), cardiac dimensions (aortic root, left atrial diameter, and left ventricular parameters) and ejection fraction (64.77 ± 2.29% vs. 65.07 ± 2.31%; *p* = 0.476) were similar. Trace to mild mitral regurgitation was detected in 10 patients (16.7%) in the ASA group and 6 subjects (10.0%) in the control group, showing no statistically significant difference (*p* = 0.287).

### 3.3. Psychometric Questionnaire Outcomes

Individuals diagnosed with an atrial septal aneurysm demonstrated substantially greater symptom burdens across all administered psychological assessments (Table 4, Supplementary Figure S2). Specifically, the state anxiety evaluation revealed notably higher averages within the aneurysm cohort (44.97 ± 9.15) compared to control subjects (32.53 ± 8.24; *p* < 0.001). A similar significant pattern emerged regarding persistent trait anxiety, where the ASA group scored considerably above the non-ASA outpatients (49.37 ± 7.22 versus 34.72 ± 10.55; *p* < 0.001). Furthermore, evaluations of depressive symptoms via the BDI illustrated a more pronounced psychological distress profile among those with the septal anomaly (19.66 ± 9.79 versus 10.07 ± 6.81; *p* < 0.001).

### 3.4. Multicollinearity and Logistic Regression Analysis

**Figure 3:**
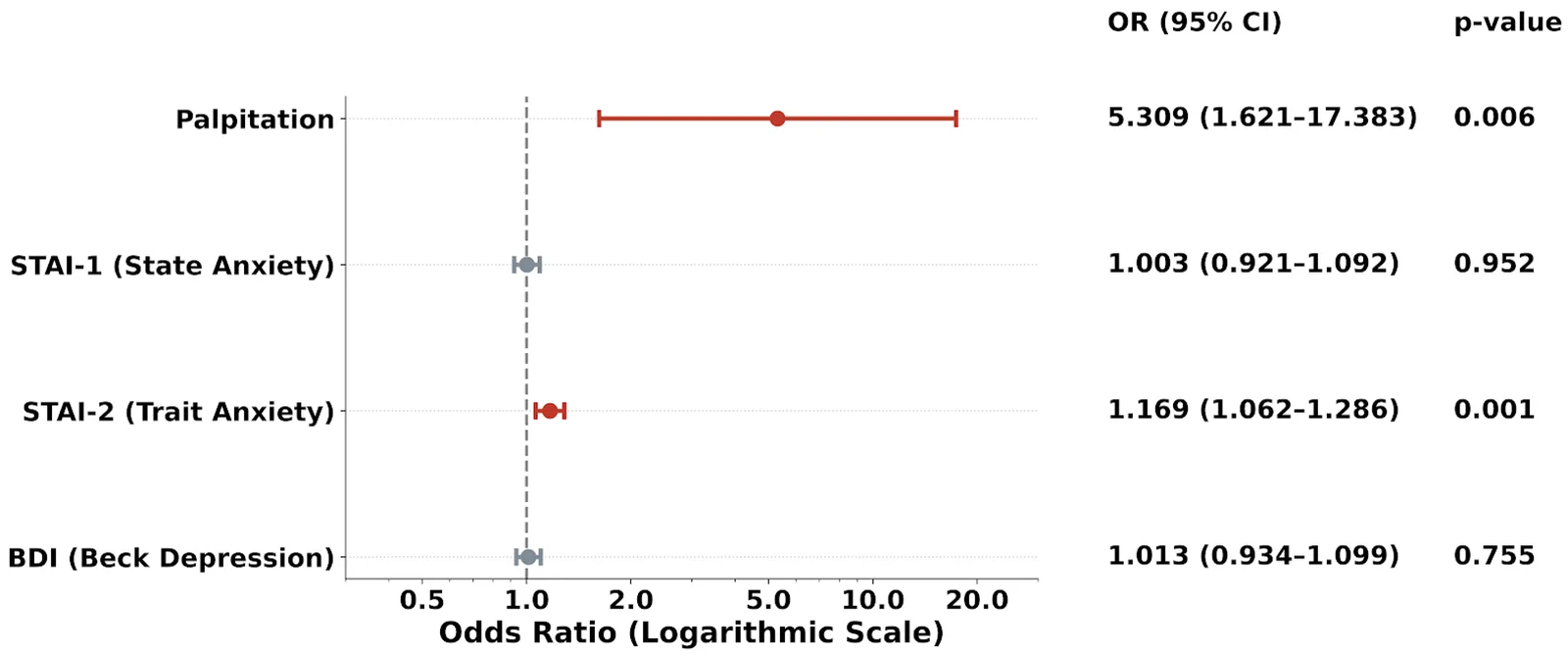
Logistic regression analysis identifying independently associated factors of atrial septal aneurysm (ASA) as the dependent variable. The forest plot presents adjusted Odds Ratios (ORs) and 95% Confidence Intervals (CIs) represented by horizontal error bars on a logarithmic scale. The overall model demonstrated an adequate fit (Hosmer–Lemeshow goodness-of-fit test, *p* = 0.38; Nagelkerke *R*^2^ = 0.34). The presence of clinical palpitations and higher trait anxiety scores (STAI-2, evaluated per one-point increment) were independently associated factors for ASA. Abbreviations: ASA, atrial septal aneurysm; BDI, Beck Depression Inventory; CI, confidence interval; OR, odds ratio; STAI, State–Trait Anxiety Inventory.

Before executing the logistic regression, we confirmed that the VIF metrics for STAI-1, STAI-2, and the BDI were 3.383, 3.705, and 2.019, respectively. For statistical transparency, Pearson correlation analysis demonstrated expected moderate-to-strong pairwise correlations among these conceptually overlapping constructs: STAI-1 and STAI-2 (r = 0.832, *p* <0.001), STAI-1 and BDI (r = 0.659, *p* < 0.001), and STAI-2 and BDI (r = 0.695, *p* < 0.001). Because all VIF parameters remained under the initially designated threshold of 5, we validated their concurrent use in the statistical model; however, we acknowledge that some stringent methodological sources recommend a stricter VIF cutoff (e.g., <2.5) for such highly correlated psychometric scales, necessitating appropriate caution in interpretation. We subsequently applied logistic regression to uncover the independent variables associated with an ASA diagnosis (Table 5, Figure 3). The overall model showed an adequate fit (Hosmer–Lemeshow goodness-of-fit test, *p* = 0.38; Nagelkerke R^2^ = 0.34). Based on this analysis, the clinical manifestation of palpitations proved to be a strong independent factor (OR: 5.309; 95% CI: 1.621–17.383; *p* = 0.006). Additionally, the trait anxiety level retained its independent predictive value, revealing that for every single-point rise in the STAI-2 score, the likelihood of detecting an ASA increased significantly (OR: 1.169 per one-point increment; 95% CI: 1.062–1.286; *p* = 0.001).

## 4. Discussion

A relationship between structural heart disease and mood disorders, namely anxiety and depressive symptoms, has been well documented within the context of atherosclerotic heart disease, heart failure, and arrhythmias [16,17,18,19]. However, the situation becomes more complicated and obscure when there is an apparently mild, clinically silent, or relatively benign structural heart disease. Past research has linked higher anxiety and depressive symptoms to conditions such as mitral valve prolapse (MVP), slow coronary flow, and coronary artery ectasia [7,8,9]. Our findings align with patterns observed in related structural conditions, such as MVP, where psychological symptom severity is elevated, despite limited and negligible hemodynamic changes. In the present study, both the Beck Depression Inventory (BDI)—a self-report rating inventory measuring characteristic attitudes and symptoms of depression—and the State–Trait Anxiety Inventory (STAI)—measuring state and trait anxiety—were found to be significantly higher in patients with ASA compared to those without ASA. Moreover, to the best of our knowledge, we have documented for the first time that ASA, characterized by localized bulging of the interatrial septum and often considered a benign incidental finding, is significantly and independently associated with clinical palpitations and trait anxiety scores measured by the STAI-2. Although ASA is generally considered a clinically silent cardiac pathology, it is reasonable to expect an association with palpitations due to its proarrhythmic potential. Patients with ASA are known to be prone to arrhythmias, either due to the tethering effect induced by the redundant atrial septal tissue or due to the structural vulnerability of the atrial myocardium itself [20]. Given that ASA is associated with cardiac arrhythmias, such as atrial fibrillation, premature atrial or ventricular complexes, and supraventricular tachycardias, a baseline level of palpitations is physiologically plausible [21,22]. However, it is worth noting that the disproportionate anxious state cannot be entirely attributed to true arrhythmogenesis. Within this context, somatosensory amplification—a psychosomatic phenomenon characterized by heightened interoceptive awareness—emerges as a fascinating proposal for the independent association between ASA and an anxious state. As classically described by Barsky [23], individuals with elevated trait anxiety exist in a state of cardiac hypervigilance. In this state, normal physiological heartbeats, minor septal excursions, or benign sinus tachycardias are amplified and misinterpreted as distressing, irregular palpitations. Thus, the palpitations in ASA patients may frequently represent a perceptual anomaly driven by heightened interoceptive awareness rather than a purely electrical one. Beyond the relationship between palpitations and ASA through either an arrhythmia-triggered mechanism or somatosensory amplification, the independent association of ASA with the anxiety score itself requires further pathophysiologic explanation. In terms of a connection between mood disorders and cardiovascular pathology or other systemic diseases, such relationships have already been documented in MVP and hypermobility joint syndrome [24,25]. Furthermore, Talati et al. reported that the genetic profiles of probands with panic disorders or social anxiety disorders, as well as their first-degree relatives, reveal a higher likelihood of having MVP and chronic headaches [26]. The intersection of ASA and anxiety raises important questions: Does ASA contribute directly to anxiety symptoms through physiological mechanisms and autonomic nervous system modulation? Whether there is a causal propensity for an anxious state in those with ASA remains to be definitively elucidated. ASA has already been discussed as having a possible connection with generalized connective tissue abnormalities [27]. The structural and functional abnormalities residing in ASA may predispose patients to supraventricular arrhythmias, which can manifest as palpitations, and may simultaneously result in a vulnerable mood, ultimately contributing to an anxious state and the aforementioned heightened interoceptive awareness. Beyond this arrhythmia-mediated relation between ASA and palpitations, given the independent association of ASA with STAI-2, an intrinsic propensity residing in ASA patients might be a reasonable explanation awaiting support from prospective clinical evidence.

### Study Limitations

Our study has several limitations. Most importantly, the cross-sectional design fundamentally precludes the establishment of causality. Based on our data, we cannot definitively determine whether the structural presence of an ASA inherently contributes to the development of anxiety, whether pre-existing trait anxiety simply increases symptom perception and health-seeking behavior (leading to the incidental detection of an ASA), or whether shared unmeasured pathophysiological or psychological factors contribute to both conditions simultaneously. Furthermore, a significant selection bias may exist inherent in our recruitment setting; patients with elevated trait anxiety or heightened symptom perception are generally more inclined to seek specialized cardiological evaluation. This increased health-seeking behavior, particularly driven by presenting symptoms like clinical palpitations, could inadvertently enrich the outpatient pool and prompt more frequent echocardiographic evaluations. Consequently, this ascertainment bias may contribute to a higher incidental detection rate of ASA among these individuals compared to the general population. Additionally, clinical palpitations were recorded subjectively based on routine medical history rather than being quantified using a standardized scoring system for frequency, duration, or severity, which limits the granularity of our symptom analysis. Fourth, psychological evaluations relied on self-reported questionnaires (STAI and BDI) rather than formal clinical psychiatric interviews based on DSM-5 criteria. Fifth, our echocardiographic assessment was limited to standard transthoracic echocardiography (TTE). Given the well-established anatomical association between ASA and PFO, we hypothesize that the transient ischemic attacks (TIA) observed in a minor subset of our ASA cohort were likely driven by undiagnosed concomitant PFOs rather than the isolated aneurysm itself; however, without systematic TEE confirmation, this mechanism remains strictly hypothetical. Sixth, our 1:1 case–control sampling ratio prevents us from inferring the true population prevalence or the absolute probability of detecting an ASA in patients presenting with anxiety. We propose somatosensory amplification as a potential mechanism; however, because we did not directly measure it, incorporating specific somatosensory amplification scales in future prospective studies is required to directly test this hypothesis. Another limitation is the absence of formal multiple-testing corrections (e.g., Bonferroni adjustments) across the baseline univariate analyses. Although our primary findings regarding palpitations and psychometric score elevations are highly robust (*p* < 0.001) and would easily survive such corrections, the nominal *p*-values of the remaining secondary clinical comparisons should be interpreted as exploratory. Finally, it is crucial to recognize that both study cohorts were drawn from a symptomatic, clinic-referred population. Consequently, our findings strictly represent an evaluation of ASA versus non-ASA presentations among cardiology attendees, rather than a comparison against psychologically healthy, community-based individuals whose baseline anxiety scores would likely be inherently lower.

## 5. Conclusions

In conclusion, this matched cross-sectional study demonstrates that clinical palpitations and an elevated trait anxiety state (STAI-2) are significantly and independently associated factors for atrial septal aneurysm (ASA). While the cross-sectional design precludes causal inferences, the findings suggest a clinical clustering of anxiety and palpitations in patients diagnosed with ASA. We hypothesize that the pronounced symptom burden in ASA patients—especially in those without documented pathological arrhythmias—may implicate the involvement of somatosensory amplification and heightened interoceptive awareness. Consequently, we propose that this intrinsic state might drive health-seeking behavior, instinctively prompting these individuals to seek medical attention. However, these mechanisms were not directly measured in the current cohort and remain proposed explanations requiring direct testing. A holistic management approach that addresses the underlying anxiety and reassures the patient regarding the generally benign nature of the anomaly—provided there are no concomitant high-risk structural abnormalities—can optimize patient care, mitigate symptom distress, and reduce unnecessary recurrent cardiovascular testing based on individual clinical risk. Future longitudinal, multidisciplinary investigations are required to definitively elucidate these cardio-psychological interactions.

## Figures and Tables

**Table 1:** Clinical and demographic characteristics of the study population.

Parameters	Patients with ASA (*n* = 60)	Subjects Without ASA (Control) (*n* = 60)	*p*-Value
Age (years)	39.78 ± 12.03	37.43 ± 8.94	0.227
Gender (Female)	20 (33.3%)	21 (35.0%)	0.849
Hypertension	5 (8.3%)	3 (5.0%)	0.468
Diabetes Mellitus	3 (5.0%)	1 (1.7%)	0.305
Hyperlipidemia	5 (8.3%)	4 (6.7%)	0.732
Body mass index (kg/m^2^)	25.90 ± 4.19	25.23 ± 4.07	0.379
Smoking	19 (31.7%)	19 (31.7%)	1.000
Chest pain	3 (5.0%)	5 (8.3%)	0.468
Palpitation	50 (83.3%)	26 (43.3%)	<0.001
Dyspnea	8 (13.3%)	6 (10.0%)	0.573
History of TIA/Amaurosis fugax	2 (3.3%)	0 (0.0%)	0.496
History of migraine	1 (1.7%)	2 (3.3%)	0.563

Data are presented as mean ± standard deviation or number (percentage). The *p*-values were calculated using an independent Student’s *t*-test for continuous variables and Chi-square or Fisher’s exact test for categorical variables. ASA: Atrial septal aneurysm; TIA: Transient ischemic attack.

**Table 2:** Electrocardiographic findings of the study population.

Parameters	Patients with ASA	Subjects Without ASA	*p*-Value
Heart rate (bpm)	77.17 ± 12.54	76.30 ± 11.19	0.691
PR interval (ms)	147.09 ± 19.15	145.65 ± 20.88	0.698
QRS duration (ms)	90.24 ± 9.09	87.62 ± 7.87	0.096
QT interval (ms)	360.83 ± 22.55	361.92 ± 23.53	0.798
QTc interval (ms)	389.10 ± 21.45	392.33 ± 23.37	0.436
Premature ventricular complex (VPC)	1 (1.7%)	0 (0.0%)	0.311
Premature atrial complex (APC)	0 (0.0%)	0 (0.0%)	N/A
Left bundle branch block (LBBB)	0 (0.0%)	0 (0.0%)	N/A
Right bundle branch block (RBBB)	1 (1.7%)	0 (0.0%)	0.311

Data are presented as mean ± standard deviation or number (percentage). The *p*-values were calculated using an independent Student’s *t*-test for continuous variables and Chi-square or Fisher’s exact test for categorical variables. ASA: Atrial septal aneurysm; APC: Premature atrial complex; LBBB: Left bundle branch block; RBBB: Right bundle branch block; VPC: Premature ventricular complex.

**Table 3:** Transthoracic echocardiographic parameters of the study groups.

Parameters	Patients with ASA (*n* = 60)	Subjects Without ASA (Control) (*n* = 60)	*p*-Value
Aortic root diameter (mm)	30.17 ± 4.19	29.95 ± 3.30	0.753
Left atrial diameter (mm)	34.63 ± 4.08	34.00 ± 3.49	0.363
LVEDD (mm)	44.35 ± 3.65	44.75 ± 3.96	0.566
LVESD (mm)	27.22 ± 2.66	27.32 ± 2.91	0.845
Ejection fraction (%)	64.77 ± 2.29	65.07 ± 2.31	0.476
Mitral regurgitation (Trace/Mild)	10 (16.7%)	6 (10.0%)	0.287

Data are presented as mean ± standard deviation or number (percentage). The *p*-values were calculated using independent Student’s *t*-test for continuous variables and Chi-square or Fisher’s exact test for categorical variables. ASA: Atrial septal aneurysm; LVEDD: Left ventricular end-diastolic diameter; LVESD: Left ventricular end-systolic diameter.

**Table 4:** Comparison of psychometric assessment profiles.

Parameters	Patients with ASA	Subjects Without ASA	*p*-Value
STAI-1 (State Anxiety)	44.97 ± 9.15	32.53 ± 8.24	<0.001
STAI-2 (Trait Anxiety)	49.37 ± 7.22	34.72 ± 10.55	<0.001
BDI (Beck Depression Inventory)	19.66 ± 9.79	10.07 ± 6.81	<0.001

Data are presented as mean ± standard deviation. The *p*-values were calculated using an independent Student’s *t*-test. ASA: Atrial septal aneurysm; BDI: Beck Depression Inventory; STAI-1: State–Trait Anxiety Inventory-1 (State Anxiety); STAI-2: State–Trait Anxiety Inventory-2 (Trait Anxiety).

**Table 5:** Logistic regression analysis for independently associated factors of ASA.

Variable	Odds Ratio (OR)	95% Confidence Interval (CI)	*p*-Value
Palpitation	5.309	1.621–17.383	0.006
STAI-1 (State Anxiety)	1.003	0.921–1.092	0.952
STAI-2 (Trait Anxiety)	1.169	1.062–1.286	0.001
Beck Depression Inventory (BDI)	1.013	0.934–1.099	0.755

The *p*-values and Odds Ratios (ORs) with 95% Confidence Intervals (CIs) were derived from logistic regression analysis. BDI: Beck Depression Inventory; CI: Confidence Interval; OR: Odds Ratio; STAI-1: State–Trait Anxiety Inventory-1 (State Anxiety); STAI-2: State–Trait Anxiety Inventory-2 (Trait Anxiety); ASA: Atrial septal aneurysm.

## Data Availability

The datasets used and/or analyzed during the current study are available from the corresponding author on reasonable request.

## Supplementary Materials

The following supporting information can be downloaded at: https://www.mdpi.com/article/10.3390/jcm15208046/s1, Figure S1. Comparison of baseline clinical and demographic characteristics between patients with atrial septal aneurysm (ASA) and non-ASA control subjects. (**A**) Anthropometric data including mean age and body mass index (BMI); error bars represent standard deviations (SD). (**B**) Prevalence of cardiovascular clinical risk factors. (**C**) Distribution of presenting clinical symptoms. Abbreviations: ASA, atrial septal aneurysm; BMI, body mass index; SD, standard deviation. Figure S2. Comparison of psychometric assessment profiles between the study groups. The bar charts illustrate the mean scores for state anxiety (STAI-1), trait anxiety (STAI-2), and depression (BDI). Error bars represent standard deviations (SD). Patients with atrial septal aneurysm (ASA) exhibited significantly higher scores across all three psychometric scales compared to the control group (*p* < 0.001 for all comparisons). Abbreviations: ASA, atrial septal aneurysm; BDI, Beck Depression Inventory; STAI, State–Trait Anxiety Inventory.

## Abbreviations

The following abbreviations are used in this manuscript:

AF	Atrial Fibrillation
ASA	Atrial Septal Aneurysm
ASD	Atrial Septal Defect
BDI	Beck Depression Inventory
BMI	Body Mass Index
CI	Confidence Interval
COPD	Chronic Obstructive Pulmonary Disease
ECG	Electrocardiography
LA	Left Atrial
LVEDD	Left Ventricular End-Diastolic Diameter
LVEF	Left Ventricular Ejection Fraction
MR	Mitral Regurgitation
MVP	Mitral Valve Prolapse
OR	Odds Ratio
PFO	Patent Foramen Ovale
SD	Standard Deviation
STAI-1	State–Trait Anxiety Inventory-1 (State Anxiety)
STAI-2	State–Trait Anxiety Inventory-2 (Trait Anxiety)
TEE	Transesophageal Echocardiography
TIA	Transient Ischemic Attack
TTE	Transthoracic Echocardiography
VIF	Variance Inflation Factor
