# Supplementary material for: Atrial Septal Aneurysm and Anxiety: A Matched Cross-Sectional Study with Prospective Enrollment Exploring Clinical and Psychosomatic Associations
Source: J Clin Med. 2026 Oct 16;15(20):8046. doi: 10.3390/jcm15208046 (PMC13705907; doi:10.3390/jcm15208046)
Supplement: Supplementary file 1 [file jcm-15-08046-s001.zip › jcm-4623697-supplementary.pdf]

Title: Atrial Septal Aneurysm and Anxiety: A Matched Cross-Sectional Study with Prospective Enrollment Exploring Clinical and Psychosomatic Associations

Authors: Doğaç Çağlar Gürbüz et al.

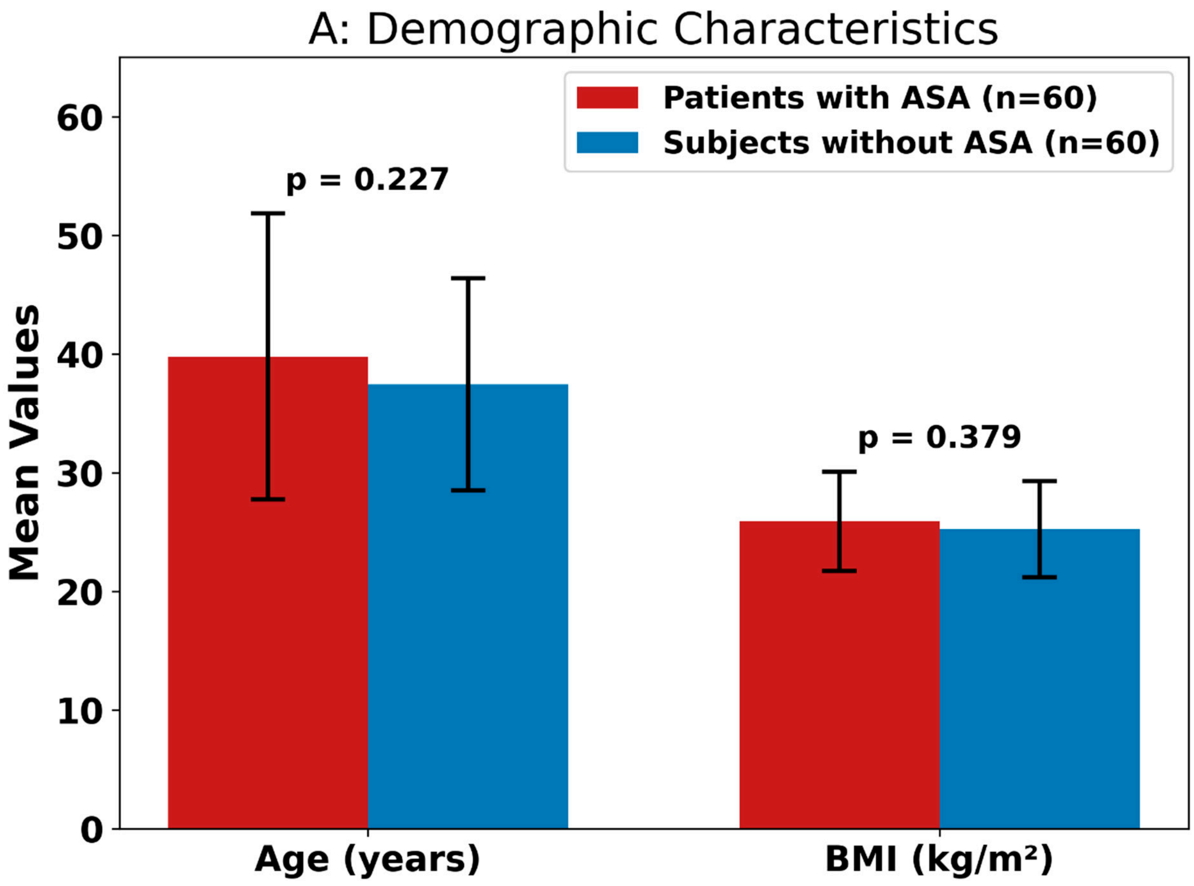

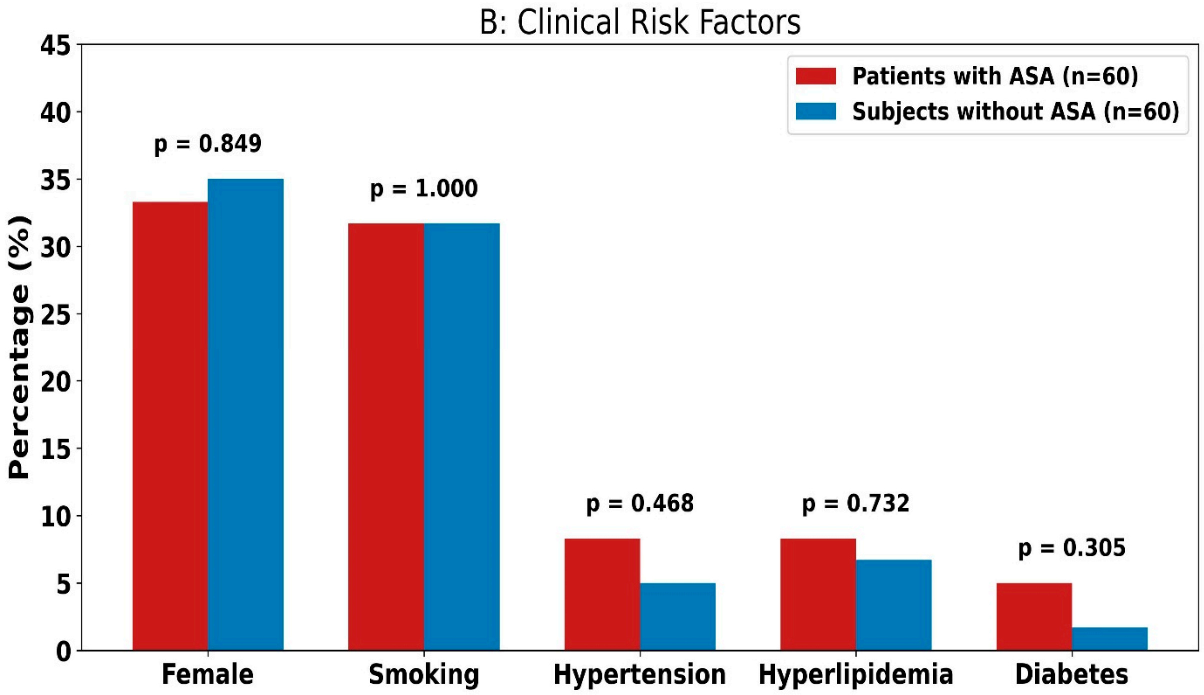

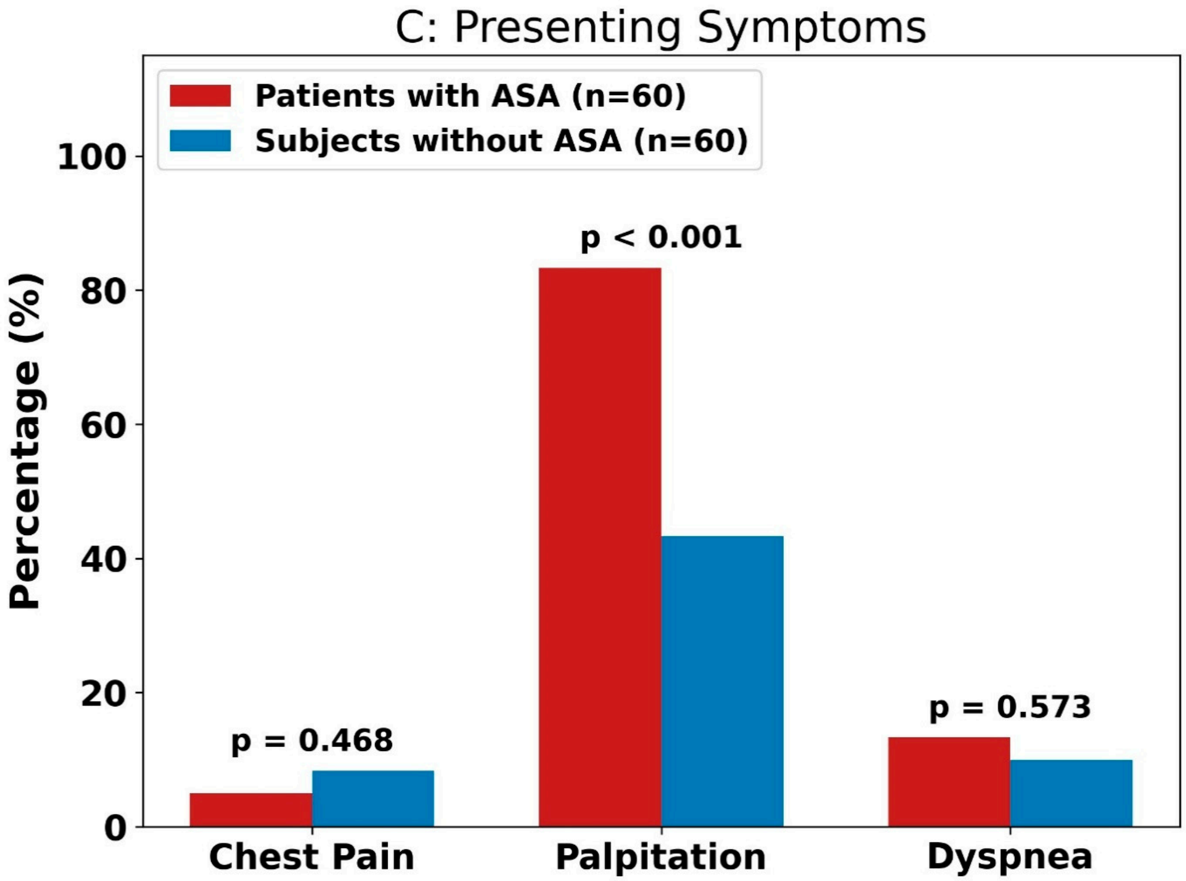

Supplementary Figure S1. *Comparison of baseline clinical and demographic characteristics between patients with atrial septal aneurysm (ASA) and non-ASA control subjects.* ***(A)*** *Anthropometric data including mean age and body mass index (BMI); error bars represent standard deviations (SD).* ***(B)*** *Prevalence of cardiovascular clinical risk factors.* ***(C)*** *Distribution of presenting clinical symptoms. Abbreviations: ASA, atrial septal aneurysm; BMI, body mass index; SD, standard deviation.*

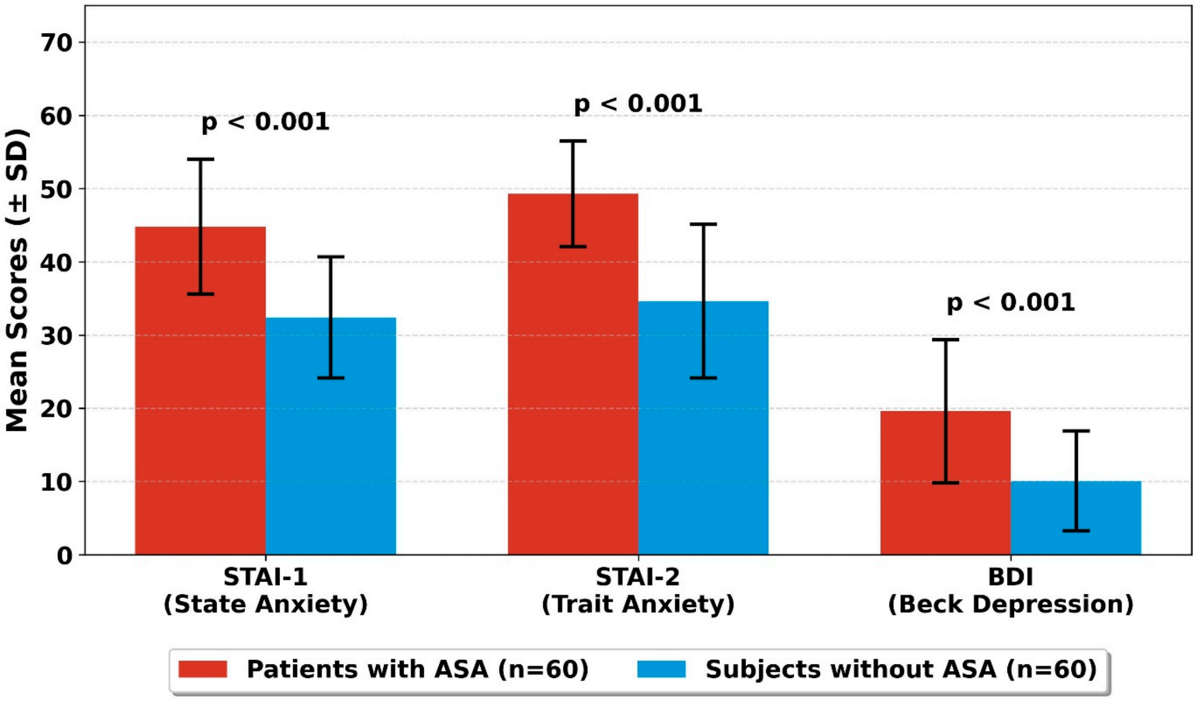

Supplementary Figure S2. *Comparison of psychometric assessment profiles between the study groups. The bar charts illustrate the mean scores for state anxiety (STAI-1), trait anxiety (STAI-2), and depression (BDI). Error bars represent standard deviations (SD). Patients with atrial septal aneurysm (ASA) exhibited significantly higher scores across all three psychometric scales compared to the control group (p <0.001 for all comparisons). Abbreviations: ASA, atrial septal aneurysm; BDI, Beck Depression Inventory; STAI, State–Trait Anxiety Inventory.*
